# SGLT2 Inhibitors Mitigate Contrast-Induced Acute Kidney Injury in Diabetes: Clinical and Experimental Evidence

**DOI:** 10.3390/ijms27041684

**Published:** 2026-02-09

**Authors:** Mu-Chi Chung, Yu-Teng Chang, Yi-Jia Guo, Chi-Jung Chung, Laing-You Wu, Ming-Ju Wu, Jeng-Jer Shieh

**Affiliations:** 1 Division of Nephrology, Department of Medicine, Taichung Veterans General Hospital, Taichung 407219, Taiwan; mcchung0322@gmail.com (M.-C.C.); t0953337796@yahoo.com.tw (Y.-T.C.); 2 Ph.D. Program in Translational Medicine, National Chung Hsing University, Taichung 402204, Taiwan; 3 Rong Hsing Research Center for Translational Medicine, National Chung Hsing University, Taichung 402204, Taiwan; 4 Division of Clinical Toxicology, Department of Medical Toxicology, Taichung Veterans General Hospital, Taichung 407219, Taiwan; 5 Department of Post-Baccalaureate Medicine, College of Medicine, National Chung Hsing University, Taichung 402204, Taiwan; 6 Department of Medical Research, Taichung Veterans General Hospital, Taichung 407219, Taiwan; 7 Institute of Biomedical Sciences, National Chung Hsing University, Taichung 402202, Taiwan; paggie0216@gmail.com; 8 Department of Public Health, College of Public Health, China Medical University, Taichung 406040, Taiwan; cjchung@mail.cmu.edu.tw (C.-J.C.); louie60550@cmu.edu.tw (L.-Y.W.); 9 Department of Medical Research, China Medical University Hospital, Taichung 406040, Taiwan; 10 School of Medicine, Chung Shan Medical University, Taichung 402306, Taiwan; 11 Graduate Institute of Biomedical Sciences, School of Medicine, College of Medicine, China Medical University, Taichung 406040, Taiwan; 12 Graduate Institute of Chinese Medicine and Drug Development, National Chung Hsing University, Taichung 402204, Taiwan

**Keywords:** SGLT2 inhibitors, contrast, acute kidney injury, dapagliflozin, type 2 diabetes mellitus

## Abstract

Contrast-induced acute kidney injury (CIAKI) is a common complication after percutaneous coronary intervention (PCI) in type 2 diabetes mellitus (T2DM). The protective role of sodium-glucose co-transporter 2 inhibitors (SGLT2i) against CIAKI remains unclear. We aimed to evaluate the effects of SGLT2i using nationwide database analysis and experimental models. A nationwide nested case–control study using Taiwan’s National Health Insurance Research Database assessed the association between SGLT2i use and risk of post-PCI dialysis. Parallel in vitro experiments examined dapagliflozin in iopamidol-treated HK-2 cells, and in vivo studies tested dapagliflozin in diabetic rats exposed to iopamidol. Clinically, SGLT2i use significantly reduced the risk of post-PCI dialysis (adjusted OR 0.33, 95% CI 0.14–0.76; *p* = 0.0094). In vitro, dapagliflozin attenuated iopamidol-induced cytotoxicity and NLRP3 inflammasome activation in HK-2 cells. In diabetic rats, dapagliflozin improved renal function, reduced tubular injury, and suppressed inflammasome-driven inflammation. In conclusion, SGLT2i protect against CIAKI by mitigating tubular injury and inflammasome activation. These findings highlight their potential as a preventive strategy for CIAKI in T2DM patients undergoing PCI.

## 1. Introduction

Sodium/glucose co-transporter 2 inhibitors (SGLT2i) are a new class of antihyperglycemic medications by inhibition of sodium/glucose co-transporter 2 in the proximal tubule of the kidney and stimulating urinary glucose excretion [1]. Dapagliflozin was the first SGLT2i approved in the world and has shown encouraging results for improving cardiovascular and renal outcomes in clinical trials of Type 2 DM (T2DM) patient [2], proteinuria chronic kidney disease [3] and heart failure [4].

Although the US Food and Drug Administration Adverse Event Reporting System has previously warned about the association between SGLT2i and the risk of acute kidney injury (AKI), a meta-analysis of large, placebo-controlled trials demonstrated that SGLT2i not only slow the progression of kidney disease but also reduce the risk of acute kidney injury in patients with type 2 diabetes [5]. In our recent work, SGLT2i users had a 0.66-fold risk for AKI and 0.56-fold risk of AKI-Dialysis compared with DPP4i users from 104,462 patients in Taiwan National Health Insurance Research Database (NHIRD), with dapagliflozin showing the lowest associated risk [6].

T2DM is recognized as a cardiovascular disease risk equivalent, with cardiovascular mortality comprising the majority of deaths [7]. Percutaneous coronary intervention (PCI) is a widely established procedure for the management of cardiovascular diseases. Unfortunately, contrast-induced acute kidney injury (CIAKI) occurs in up to 30% of patients receiving iodinated contrast media and was considered to be the one of the most common causes of hospital-acquired AKI [8].

Previous studies have demonstrated that SGLT2i reduce the risk of CIAKI in patients with diabetes undergoing PCI [9]; however, some studies present conflicting evidence [10,11]. From a pathophysiological perspective, the effects of SGLT2i, such as their anti-apoptotic and anti-inflammatory actions on renal tubular cells [12,13], may provide protective benefits against CIAKI. In this study, we aim to evaluate the protective effects of SGLT2i against CIAKI through an integrated approach, encompassing clinical data, cell models, and animal studies.

## 2. Results

### 2.1. Post-PCI Dialysis Cases Showed a More Severe Clinical Profile and Less SGLT2i Exposure

In our study, we identified 3784 cases as post PCI dialysis and 15,136 controls (Table 1). The mean ages of all study population were approximately 70 years and 69.1% were aged ≥65. The study population consisted of more male patients (61.6%). A higher percentage of post PCI dialysis cases had comorbidities, such as hypertension, CVD, CKD, PAD, heart failure, glomerulonephritis, STEMI, NSTEMI as well as oral and injectable anti-diabetic drug usage except SGLT2i, compared to the controls. Conversely, the controls had higher usage of SGLT2i than the cases.

### 2.2. SGLT2i Use as an Independent Protective Factor for Post PCI Dialysis Risk

The results suggested a significant association between SGLT2i use and the risk of post PCI dialysis (OR = 0.35; 95% CI = 0.17–0.72, *p* = 0.0043) (Table 2). Even adjustment for age, gender, comorbidities (including hypertension, hyperlipidemia, CVD, CAD, CKD, PAD, heart failure, glomerulonephritis, STEMI, NSTEMI and unstable angina), oral anti-diabetes drug, injectable anti-diabetes drug and other medications, a protective association between SGLT2i use and risk of dialysis after contrast remained observed (OR = 0.33; 95% CI = 0.14–0.76, *p* = 0.0094). In subgroup analysis, protective associations with SGLT2i use against post PCI dialysis were significantly observed in the patients with hypertension, CKD, and without CVD, PAD, heart failure, glomerulonephritis, STEMI, NSTEMI, and unstable Angina (Table 3). The protective effects of SGLT2i appeared consistent across all subgroups (*p* for interaction > 0.05).

### 2.3. Dose-Dependent Effects of Iopamidol and Dapagliflozin on HK-2 Cell Viability and Cytotoxicity

To determine the optimal experimental conditions, we first evaluated the cytotoxicity of iopamidol under both physiological (5.5 mM) and high glucose (25 mM) conditions. Results showed that iopamidol treatment induced a dose-dependent decrease in cell viability in both glucose environments, with a comparable extent of cytotoxicity observed (IC50: 192.68 ± 13.66 mg I/mL for LG vs. 188.45 mg I/mL for HG; Figure S3A). Despite the lack of significant difference between the two glycemic conditions in this acute in vitro setting, based on established models of diabetic kidney injury [14], we selected the high-glucose model (25 mM) for all subsequent experiments. This decision was made to strictly align with our in vivo diabetic rat model and the specific clinical population (T2DM patients) under investigation. In this high-glucose environment, treatment with iopamidol (25–200 mg I/mL) for 24 h resulted in a dose-dependent decrease in cell viability. Concurrently, flow cytometry revealed an increase in the sub-G1 population, indicative of cell death, with higher iopamidol doses (Figure S3B,C). Regarding dapagliflozin, concentrations ranging from 0 to 100 μM were tested; it showed no cytotoxic effects up to 75 μM, whereas reduced viability was observed at 100 μM (Figure S3). Consequently, iopamidol at 200 mg I/mL and dapagliflozin at 75 μM under high glucose conditions were selected for subsequent experiments.

### 2.4. Dapagliflozin Attenuates Iopamidol-Induced Cytotoxicity in HK-2 Cells and Preserves Renal Structural Integrity in Diabetic Rat Models

**Figure 1:**
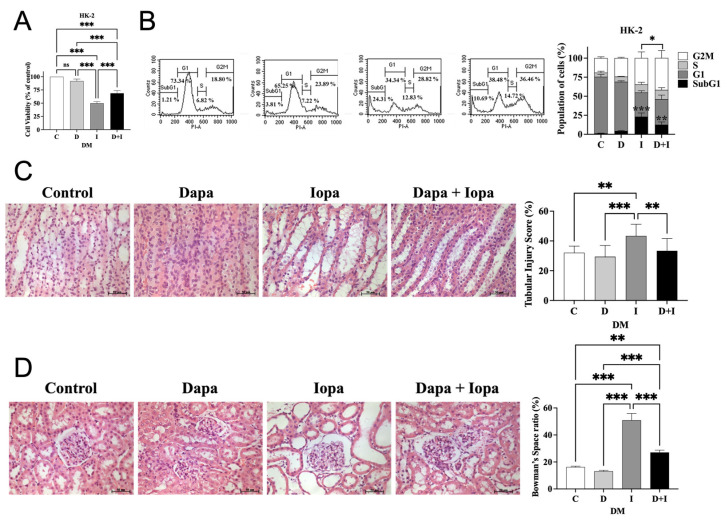
Dapagliflozin Attenuates Iopamidol-Induced Cytotoxicity in HK-2 Cells and Preserves Renal Structural Integrity in Diabetic Rat Models. (**A**) Quantification of cell viability in HK-2 cells under different treatment conditions. (**B**) Flow cytometry analysis of cell cycle distribution in HK-2 cells after treatment with different compounds. Representative histograms and corresponding quantification are shown. (**C**) Representative histological images of renal tubules stained with H&E. The bar graph represents the tubular injury score. (**D**) Quantitative analysis of glomerular morphology based on H&E staining. The bar graph represents the Bowman’s space ratio (defined as the area of Bowman’s space divided by the total glomerular area), indicating the extent of glomerular shrinkage. The group designations are as follows: C (Control), D or Dapa (Dapagliflozin), I or Iopa (Iopamidol), and D + I (Dapagliflozin + Iopamidol). All experiments were performed at least three times independently. Statistical analysis was conducted using one-way ANOVA followed by post hoc tests. Data are presented as mean ± standard deviation (SD), and statistical significance is represented as *p* < 0.05: *, *p* < 0.01: **, and *p* < 0.001: ***. Scale bars are indicated in the images.

HK-2 cells were pretreated with 75 μM dapagliflozin for 2 h, followed by exposure to iopamidol (200 mg I/mL) for 24 h. Pretreatment with dapagliflozin significantly improved cell viability (Figure 1A) and attenuated cell death, as evidenced by a decrease in the sub-G1 population (Figure 1B). In diabetic rat models, histological analysis showed that iopamidol caused severe renal tissue damage. The iopamidol-treated group (DM-I) exhibited marked tubular injury, including vacuolar degeneration, tubular casts, and luminal congestion, resulting in a significantly increased tubular injury score (Figure 1C). Furthermore, quantitative morphometric analysis of the glomeruli revealed that iopamidol treatment led to a significant expansion of the Bowman’s space, indicative of glomerular shrinkage. Importantly, dapagliflozin pretreatment effectively ameliorated these histological abnormalities. It not only reduced the tubular injury score but also preserved glomerular morphology, maintaining a normal Bowman’s space ratio (Figure 1D). Thus, dapagliflozin effectively protects against iopamidol-induced cytotoxicity in vitro and ameliorates histological markers of both tubular and glomerular injury in vivo.

### 2.5. Dapagliflozin Stabilizes Renal Function and Attenuates Iopamidol-Induced Inflammasome Activation in HK-2 Cells and Diabetic Rat Model

**Figure 2:**
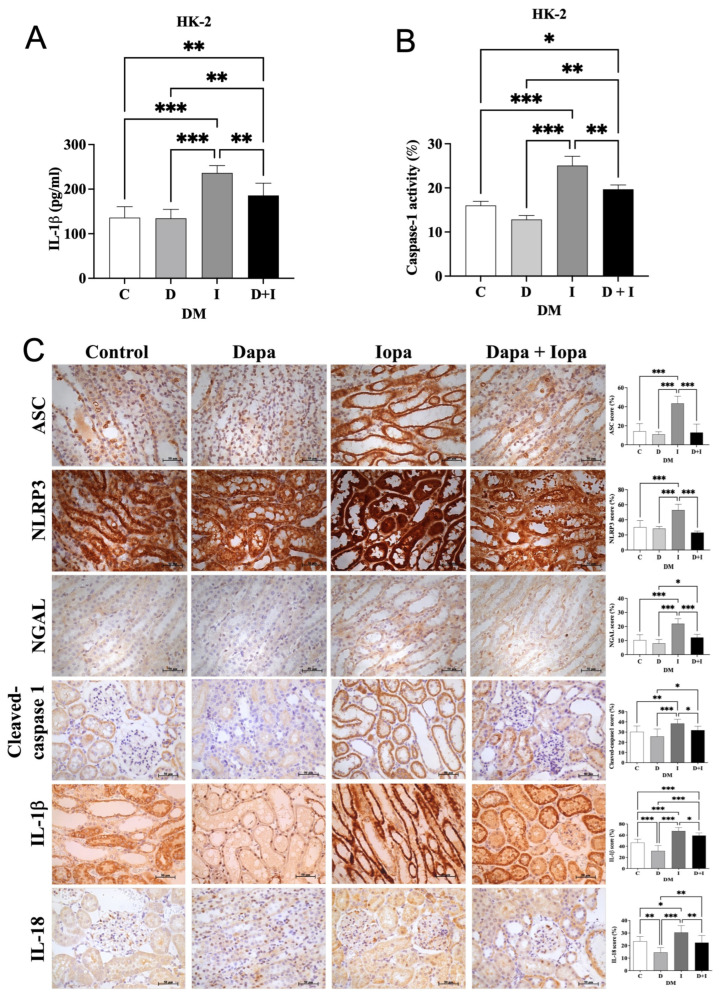
Dapagliflozin Attenuates Iopamidol-Induced Inflammasome Activation in HK-2 Cells and Diabetic Rat Model. (**A**) Quantification of IL-1β levels in HK-2 cells under different treatment conditions. (**B**) Measurement of caspase-1 activity in HK-2 cells after different treatments. (**C**) Representative immunohistochemical staining images showing the expression of ASC, NLRP3, NGAL, cleaved caspase-1, IL-1β, and IL-18 in renal tissues under different treatment conditions. The corresponding bar graphs quantify the percentage of positive staining area. For all experiments, *n* = 8 per group. The group designations are as follows: C (Control), D or Dapa (Dapagliflozin), I or Iopa (Iopamidol), and D + I (Dapagliflozin + Iopamidol). All experiments were performed at least three times independently. Statistical analysis was conducted using one-way ANOVA followed by post hoc tests. Data are presented as mean ± standard deviation (SD), and statistical significance is represented as *p* < 0.05: *, *p* < 0.01: **, and *p* < 0.001: ***.

HK-2 cells treated by iopamidol (200 mg I/mL) showed significant increases in IL-1β levels and caspase-1 activity compared to controls. Dapagliflozin pretreatment effectively reduced IL-1β levels, caspase-1 activity, and cleaved caspase-1 expression (Figure 2A,B). In diabetic rats, iopamidol induced strong renal tubular expression of ASC, NLRP3, NGAL, cleaved caspase-1, IL-1β, and IL-18, indicating NLRP3 inflammasome activation and inflammation (Figure 2C). Dapagliflozin significantly suppressed these markers, demonstrating its anti-inflammatory effects. Additionally, iopamidol significantly elevated BUN, creatinine, and urinary NGAL/creatinine levels while reducing creatinine clearance (Figure S4A–D). Body weight remained stable, and blood glucose levels showed a trend of decrease but not significant in dapagliflozin-treated groups (Figure S4E,F).

## 3. Discussion

This study is the first to integrate clinical, cellular, and animal models to investigate the relationship between SGLT2i and CIAKI. Clinically, among T2DM patients not using metformin, only SGLT2i use was associated with a reduced risk of post-PCI dialysis. In cellular and animal models, dapagliflozin attenuated contrast-induced cytotoxicity, preserved glomerular structural integrity, improved renal function, and significantly mitigated inflammasome activation.

AKI after PCI is associated with increased long-term mortality [15], and emergency dialysis post-PCI significantly raises in-hospital mortality risk [16]. Among antidiabetic drugs, only SGLTi have been studied for reducing CIAKI risk. While a meta-analysis reported a 60% reduction in CIAKI risk with SGLT2i in PCI patients [9], recent studies have shown inconsistent results, ranging from protective [17] to neutral [10,18] or even harmful effects [11]. These studies are limited by short follow-ups and often lack comparisons with other antidiabetic drugs. Our study leverages the strengths of a large-scale nationwide cohort and extends the follow-up to 30 days, allowing us to detect the significant protective effect of SGLT2i against dialysis.

SGLT2i have demonstrated protective effects against AKI across various experimental models, including those induced by sepsis [13], heart failure [19], and nephrotoxins [12]. Beyond their hemodynamic effects, mounting evidence suggests that SGLT2 inhibitors exert potent anti-inflammatory properties [20]. Empagliflozin reduces renal inflammation in septic shock models [13] and alleviates oxidative stress in diabetic rat post-myocardial infarction [19]. Recent studies have also highlighted that dapagliflozin can suppress the inflammatory cascade by inhibiting the activation of innate immune responses in the kidney [21]. These mechanisms highlight the potential of SGLT2is to mitigate AKI through anti-inflammatory and antioxidative pathways, in addition to improving renal cortical oxygenation [22] and decrease intraglomerular pressure [1], contributing to their kidney-protective effects.

CIAKI-related cellular and tubular effects involve multiple pathways. A critical mechanism is the direct toxicity of iodinated contrast media on renal tubular epithelial cells, which triggers the generation of reactive oxygen species (ROS) and subsequent inflammatory responses [23]. ER stress, mediated by the pro-apoptotic unfolded protein response (UPR) pathway, has also been implicated [24]. Autophagy is activated in contrast media-induced cytotoxicity, serving a protective role in renal tubular epithelial cells [25]. Additionally, a recent study identified the NLRP3 inflammasome as a key mediator of CIAKI [26], with tubular reabsorption of contrast media shown to trigger immune activation through resident renal phagocytes and infiltrating leukocytes, further amplifying inflammation. Contrast media can act as a danger-associated molecular pattern (DAMP), stimulating the assembly of the NLRP3 inflammasome, which leads to the maturation and release of pro-inflammatory cytokines such as IL-1β and IL-18. This inflammatory storm recruits immune cells and exacerbates tubular structural injury [27].

In our experimental models, HK-2 cells were cultured in high-glucose medium (25 mM), a widely used condition in diabetic nephropathy research that mimics poorly controlled hyperglycemia and provides a stable environment for evaluating tubular injury [14]. To further elucidate the specific role of hyperglycemia in CIAKI, we also assessed cell viability under physiological glucose concentrations (5.5 mM) as a normoglycemic control. Our findings revealed that iopamidol induced significant and comparable cytotoxicity in both normoglycemic and hyperglycemic conditions. This suggests that the intrinsic direct toxicity of the contrast medium is the dominant factor driving cell death in this acute in vitro exposure model. However, we maintained the high-glucose environment for subsequent assays to ensure the experimental design remained biologically relevant to the pathophysiology of diabetic nephropathy and consistent with our clinical focus on T2DM patients. Similarly, our streptozotocin-induced diabetic rat model, with blood glucose levels > 400 mg/dL, reflects sustained hyperglycemia and its renal consequences, following standardized protocols commonly used in nephrotoxicity studies [28]. Although the cellular and animal models of diabetic nephropathy used in this study do not fully replicate the human disease, the observed anti-inflammatory and renoprotective effects of SGLT2 inhibitors align with the protective benefits reported in clinical data.

In our study, we confirmed that contrast induces tubular injury and inflammasome activation. Meanwhile, we also demonstrated that dapagliflozin not only attenuates structural damage but also inhibits inflammasome activation, a phenomenon observed in other experimental models as well. SGLT2i have been shown to reduce NLRP3 inflammasome activation in multiple settings, including T2DM patients with high cardiovascular risk by modulating ketone and insulin metabolism [29] and in an ischemia/reperfusion-induced fibrosis model via increased tubular itaconate production [30]. Additionally, a recent review highlighted that SGLT2 inhibitors mitigate diabetic kidney disease progression by inhibiting the NLRP3 inflammasome, reducing oxidative stress, and decreasing pro-inflammatory markers [31]. These findings reinforce the anti-inflammatory and renoprotective effects of SGLT2 inhibitors, further supporting their role in preventing CIAKI.

A previous basic study on SGLT2i and CIAKI reported that dapagliflozin protects against CIAKI by inhibiting the HIF-1α/HE4/NF-κB pathway [32]. Their findings suggest that HIF-1α activation leads to increased HE4 and NF-κB expression, promoting renal tubular injury and inflammation. Dapagliflozin mitigates these effects by reducing oxygen consumption in renal tubular cells, thereby alleviating intracellular hypoxia and suppressing HIF-1α-mediated inflammatory signaling. While this study focused on hypoxia-induced injury, our findings highlight inflammasome activation as another key mechanism. Given that HIF-1α has been linked to NLRP3 inflammasome activation in other kidney injury models [33], their interplay in CIAKI warrants further investigation.

This study is the first nationwide analysis on SGLT2i and CIAKI, utilizing dialysis as a key renal outcome. It is also the first to integrate clinical and experimental data, offering a comprehensive perspective on this issue.

Several limitations should be acknowledged. First, baseline glomerular filtration rate, detailed laboratory parameters, and specific details regarding the volume and type of contrast media used could not be obtained from ICD codes, which limited our ability to accurately assess CKD severity and its contribution to post-PCI dialysis risk. However, we addressed this by adjusting for CKD diagnosis in our multivariate models and performing subgroup analyses, which showed a consistent protective effect of SGLT2i regardless of CKD status. Second, our findings are applicable only to T2DM patients not receiving metformin, as both the clinical and experimental models focused exclusively on dapagliflozin; thus, the generalizability to patients treated with metformin or other SGLT2 inhibitors remains uncertain. Third, while consistent SGLT2i protection was observed across our clinical and experimental systems, outcomes were limited to a 30-day post-PCI window, which may not capture long-term renal recovery. Additionally, several limitations should be noted, including the inherent heterogeneity between the clinical T2DM cohort and the STZ-induced T1DM rat model, as well as the lack of non-diabetic controls in the in vivo studies, which may limit the generalizability of our findings. Furthermore, the in vitro concentrations used were selected to elicit acute responses and demonstrate direct mechanistic effects, which may exceed typical clinical plasma levels; therefore, future research employing more uniform models and pharmacokinetic considerations is warranted to further refine these protective mechanisms. Fifth, although we demonstrated that dapagliflozin attenuates inflammasome activation, we did not establish whether this represents the key mediating pathway, which warrants further investigation. Finally, our in vitro results indicate that dapagliflozin exerts a direct protective effect against iopamidol-induced cytotoxicity and inflammation. However, in vivo, the observed benefits may result from either direct mechanism (as suggested by the in vitro data) or indirect mechanisms (such as glucose reduction), which requires further clarification.

## 4. Materials and Methods

### 4.1. Study Design and Population

We performed a retrospective nested case–control study in a cohort of patients with type 2 diabetes mellitus (T2DM) without end-stage renal disease (ESRD) prior to undergoing percutaneous coronary intervention (PCI) between 1 May 2016 (the release date of SGLT2 inhibitors in Taiwan), and 31 December 2021. All participants were aged 18 years or older, and their data were retrieved from the Taiwan National Health Insurance Research Database (NHIRD). Patients with T2DM were identified using the International Classification of Diseases, 9th and 10th Revision, Clinical Modification (ICD-9-CM and ICD-10-CM) codes, defined as having at least three outpatient visits or one hospitalization with a diagnosis of T2DM within one year. ESRD was defined according to the Registry for Catastrophic Illness Patient Database. This study was approved by the Research Ethics Committee of China Medical University Hospital (CMUH109-REC1-018) and conducted in accordance with the principles of the Declaration of Helsinki. The requirement for informed consent was waived because all data were de-identified in the NHIRD before analysis.

### 4.2. Dialysis Subjects and Matched Controls

We excluded all patients with a prescription for metformin within 90 days prior to the PCI procedure, as some guidelines still recommend temporarily discontinuing metformin before contrast agent administration [34,35]. To eliminate this potential confounding factor, we excluded this patient group. A total of 3785 T2DM patients, who received PCI procedure (33076A, 33076B, 33077A, 33077B, 33078A, 33078B) and then initiated dialysis treatment within 30 days, were identified as our cases, post PCI dialysis group. The control group consisted of the remaining 40,485 patients who underwent PCI without requiring acute dialysis. The controls were then frequency matched for age, sex, the year of receiving PCI and incident dialysis, with the cases at a 1:4 ratio. The study protocol is shown in Figure S1.

### 4.3. SGLT2i, Other Diabetic Medications, and Covariates

We evaluated the potential impact of anti-diabetic drugs by class: SGLT2i, other oral anti-diabetic drugs and injectable anti-diabetic drugs. All explored medication included SGLT2i, DPP4 inhibitors, GLP-1 receptor agonists, insulin, alpha-glucosidase inhibitors, sulfonylureas, thiazolidinediones, and meglitinides, along with other medications like statins, aspirin, ACE inhibitors (ACEIs), and angiotensin receptor blockers (ARBs). Medication usage was ascertained based on the duration from prior to receiving PCI 90 days. In addition, comorbidities included hypertension, hyperlipidemia, cerebral vascular disease (CVD), coronary artery disease (CAD), chronic kidney disease (CKD), peripheral artery disease (PAD), heart failure, glomerulonephritis, STEMI (ST elevation myocardial infraction), NSTEMI (Non ST elevation myocardial infraction), and unstable angina were defined within a year before the incidence of dialysis.

### 4.4. Cell Culture and Drug Treatment

Human renal proximal tubule epithelial cells (HK-2 cells) were obtained from the Food Industry Research and Development Institute (FIRDI, Hsinchu, Taiwan). The cells were cultured in keratinocyte serum-free medium (K-SFM; Gibco, Thermo Fisher Scientific, Waltham, MA, USA) supplemented with 0.05 mg/mL bovine pituitary extract (BPE), 5 ng/mL recombinant human epidermal growth factor (EGF), and 5 μg/mL gentamicin (Gibco, Thermo Fisher Scientific). The cultures were maintained at 37 °C in a humidified atmosphere containing 5% CO_2_. HK-2 cells were treated with iopamidol, obtained from Bracco Diagnostics (Princeton, NJ, USA), and/or dapagliflozin, procured from Selleckchem (Houston, TX, USA).

### 4.5. Cell Viability and DNA Content Assay

HK-2 cells were seeded in 6-well plates and treated with iopamidol, dapagliflozin, or both (dapagliflozin as a 1 h pre-treatment). After 24 h, cell viability was assessed using trypan blue staining. For cell cycle analysis, cells were fixed in 75% ethanol, stained with propidium iodide (Thermo Fisher Scientific), and analyzed using a Cytomics™ FC500 Flow Cytometer (Beckman Coulter, Taipei, Taiwan).

### 4.6. Enzyme-Linked Immunosorbent Assay (ELISA) and Caspase1 Activity Assay

IL-1β levels in HK-2 cell supernatants were measured using the Human IL-1β ELISA Kit (ABclonal Inc., Woburn, MA, USA), and caspase-1 activity was assessed with the Caspase-1 (Active) Staining Kit—Green Fluorescence (Abcam, Cambridge, UK), following the manufacturers’ protocols.

### 4.7. Animals and In Vivo Experiments

Six-week-old male Sprague-Dawley (SD) rats were obtained from BioLASCO Taiwan (Taipei, Taiwan). Diabetes was induced using the STZ model [28]. Contrast-induced nephropathy (CIN) was induced after 16 h of fasting by femoral vein injection of indomethacin (10 mg/kg), N-nitro-L-arginine methyl ester (10 mg/kg), and iopamidol (2.9 g iodine/kg) [36]. All chemicals, including STZ, indomethacin, N-nitro-L-arginine methyl ester, and iopamidol, were purchased from Sigma-Aldrich (St. Louis, MO, USA). For the control group, saline was administered instead of iopamidol.

Thirty-two rats were randomly assigned to four groups (*n* = 8 per group): control (DM-C, saline injections during CIN procedures), dapagliflozin-treated (DM-D, received 10 mg/kg dapagliflozin orally for 7 days followed by saline injections), iopamidol-treated (DM-I, underwent CIN induction without prior dapagliflozin treatment), and dapagliflozin + iopamidol-treated (DM-DI, received dapagliflozin for 7 days prior to CIN induction). The overall experimental timeline and grouping strategy are illustrated in Figure S2, which outlines the induction of diabetes, drug treatment, contrast administration, and sample collection schedule.

Post-operation, rats were housed in metabolic cages for 72 h to monitor fluid and food intake. At the end of the experiments, all rats were euthanized using CO_2_ inhalation in a chamber at a displacement rate of 30–70% of the chamber volume per minute (7.2 L/min for rats), until respiration and heartbeat ceased. This procedure was performed in accordance with the American Veterinary Medical Association (AVMA) Guidelines for the Euthanasia of Animals (2020). All procedures were performed following institutional ethical guidelines and approved by the Animal Care and Research Committee of Taichung Veterans General Hospital (permit number: La-1101800).

### 4.8. Renal Function, Cytokine Analysis and Tissue Analysis

Blood samples were collected from the left femoral artery, and serum was separated by centrifugation at 1200× *g* for 10 min at 4 °C. Serum and urinary creatinine levels, as well as urinary NGAL levels, were measured using ELISA kits. Urinary NGAL was assessed with a Rat Lipocalin-2 ELISA Kit (Abcam), while creatinine levels were determined using a Rat Creatinine ELISA Kit (MyBioSource, San Diego, CA, USA). Kidneys were excised for biochemical, histopathological, and immunohistochemical analyses.

Renal tissues were fixed in 4% formalin, embedded in paraffin, and sectioned at 4 μm. Histopathology was evaluated using H&E staining, Tubular injury was quantified as the percentage of tubules displaying damage (including vacuolar degeneration, tubular casts, and luminal congestion) in at least 20 randomly selected fields per section. Additionally, glomerular morphometry was performed to assess structural integrity. Digital images of the renal cortex were analyzed to measure the cross-sectional area of the glomerular tuft (*A_tuft_*) and the total area enclosed by the Bowman’s capsule (*A_capsule_*). The Bowman’s space ratio was calculated using the formula: (*A_capsule_* − *A_tuft_*)/*A_capsule_*. At least 20 randomly selected glomeruli were analyzed per animal in a blinded manner.

### 4.9. Immunohistochemistry

Paraffin-embedded renal tissues were sectioned at 4 μm thickness. Sections were deparaffinized in xylene and rehydrated through a graded ethanol series. Antigen retrieval was performed by heating the sections in 0.01 M citrate buffer (pH 6.0) for 20 min. To block endogenous peroxidase activity, sections were treated with 3% hydrogen peroxide for 10 min, followed by blocking with 5% bovine serum albumin (BSA) to prevent non-specific binding. The sections were then incubated overnight at 4 °C with the following primary antibodies: anti-NLRP3 (1:200, iReal, Hsinchu, Taiwan), anti-ASC (1:200, iReal), anti-cleaved caspase-1 (1:200, iReal), anti-IL-1β (1:200, iReal), and anti-IL-18 (1:100, GeneTex, Irvine, CA, USA). After washing, the sections were incubated with a horseradish peroxidase (HRP)-conjugated secondary antibody for 1 h at room temperature. Immunoreactivity was visualized using a 3,3′-diaminobenzidine (DAB) substrate kit, and nuclei were counterstained with hematoxylin. Finally, sections were dehydrated and mounted. The percentage of positive staining area was quantified using NIS-elements BR software 4.0.

### 4.10. Statistical Analysis

For NHIRD studies, continuous variables are presented as mean ± SD, and categorical variables as numbers with percentages. Group differences were analyzed using the Mann–Whitney U test for continuous variables and Chi-square tests for categorical variables. Logistic regression analysis (univariate and multivariate) was used to evaluate the association between SGLT2i/other anti-diabetic drugs and the risk of post-PCI dialysis, adjusting for confounders, with odds ratios (ORs) with corresponding 95% confidence intervals (CIs) reported. Stratified analyses by comorbidities and medication use were also performed. Statistical analyses were conducted using statistical software version 9.4 (SAS Institute), with significance set at *p* < 0.05. For in vivo and in vitro experiments, at least three independent replicates were conducted. Assays were performed in duplicate or triplicate. Statistical differences were analyzed using One-way ANOVA, with significance set at *p* < 0.05.

## 5. Conclusions

SGLT2 inhibitors were associated with a reduced risk of post-PCI dialysis and protected against CIAKI by mitigating renal structural injury and inhibiting NLRP3 inflammasome activation. These findings support the potential renoprotective role of SGLT2i through anti-inflammatory pathways, warranting further investigation into detailed mechanisms.

## Figures and Tables

**Table 1:** Clinical Characteristics of Frequency-Matched T2DM patients by Dialysis Status Within 30 Days Post-PCI.

	Overall	Post PCI Dialysis Group	Non-Dialysis Group	
	(N = 18,920)	(N = 3784)	(N = 15,136)	*p* Value
Age, Mean ± Std	69.88 ± 11.49	69.99 ± 11.52	69.86 ± 11.48	0.5054
Stratify age, *n* (%)				1
26–34	15 (0.08%)	3 (0.08%)	12 (0.08%)	
35–44	370 (1.96%)	74 (1.96%)	296 (1.96%)	
45–54	1485 (7.85%)	297 (7.85%)	1188 (7.85%)	
55–64	3975 (21.01%)	795 (21.01%)	3180 (21.01%)	
65–74	5945 (31.42%)	1189 (31.42%)	4756 (31.42%)	
75–84	5250 (27.75%)	1050 (27.75%)	4200 (27.75%)	
≥85	1880 (9.94%)	376 (9.94%)	1504 (9.94%)	
Gender (Female), *n* (%)	7275 (38.45%)	1455 (38.45%)	5820 (38.45%)	1
Comorbidities				
Hypertension	14,743 (77.92%)	3133 (82.80%)	11,610 (76.70%)	<0.0001
Hyperlipidemia	9869 (52.16%)	1699 (44.90%)	8170 (53.98%)	<0.0001
Cerebral Vascular Disease	2336 (12.35%)	513 (13.56%)	1823 (12.04%)	0.0114
Coronary Artery Disease	9190 (48.57%)	1307 (34.54%)	7883 (52.08%)	<0.0001
Chronic Kidney Disease	6186 (32.70%)	2752 (72.73%)	3434 (22.69%)	<0.0001
Peripheral Artery Disease	574 (3.03%)	145 (3.83%)	429 (2.83%)	0.0014
Heart Failure	3198 (16.90%)	1053 (27.83%)	2145 (14.17%)	<0.0001
Glomerulonephritis	659 (3.48%)	296 (7.82%)	363 (2.40%)	<0.0001
STEMI	328 (1.73%)	84 (2.22%)	244 (1.61%)	0.0104
NSTEMI	747 (3.95%)	238 (6.29%)	509 (3.36%)	<0.0001
Unstable Angina	810 (4.28%)	125 (3.30%)	685 (4.53%)	0.0009
DM medications				
SGLT2i	99 (0.52%)	8 (0.21%)	91 (0.60%)	0.003
DPP4i	430 (2.27%)	114 (3.01%)	316 (2.09%)	0.0006
GLP-1	34 (0.18%)	10 (0.26%)	24 (0.16%)	0.1697
Insulin	1104 (5.84%)	414 (10.94%)	690 (4.56%)	<0.0001
Alpha glucosidase inhibitors	886 (4.68%)	217 (5.73%)	669 (4.42%)	0.0006
Sulfonylureas	389 (2.06%)	83 (2.19%)	306 (2.02%)	0.5054
Thiazolidinediones	242 (1.28%)	63 (1.66%)	179 (1.18%)	0.0182
Meglitinides	399 (2.11%)	127 (3.36%)	272 (1.80%)	<0.0001
Other medications				
Statin	1681 (8.88%)	474 (12.53%)	1207 (7.97%)	<0.0001
Aspirin	1218 (6.44%)	294 (7.77%)	924 (6.10%)	0.0002
ACEI	129 (0.68%)	29 (0.77%)	100 (0.66%)	0.4797
ARB	2014 (10.64%)	466 (12.32%)	1548 (10.23%)	0.0002

**Table 2:** Adjusted and Crude Odds Ratios for Dialysis Risk Within 30 Days in Frequency-Matched T2DM patients, Stratified by Antidiabetic Medication Use.

	Crude	Model 1	Model 2	Model 3
	OR (95% CI)	OR (95% CI)	OR (95% CI)	OR (95% CI)
SGLT2i	0.35 (0.17–0.72) **	0.35 (0.17–0.72) **	0.38 (0.17–0.87) *	0.33 (0.14–0.76) **
DPP4i	1.46 (1.17–1.81) **	1.45 (1.17–1.80) **	1.00 (0.77–1.30)	0.87 (0.66–1.14)
GLP-1 agonist	1.67 (0.80–3.49)	1.67 (0.80–3.50)	0.99 (0.41–2.38)	0.72 (0.29–1.78)
Insulin	2.59 (2.28–2.94) ***	2.58 (2.27–2.94) ***	1.70 (1.46–1.99) ***	1.66 (1.39–1.97) ***
Alpha glucosidase inhibitors	1.32 (1.13–1.55) **	1.31 (1.12–1.54) **	1.10 (0.91–1.33)	1.08 (0.87–1.34)
Sulfonylureas	1.09 (0.85–1.39)	1.08 (0.85–1.38)	0.91 (0.68–1.21)	0.83 (0.61–1.13)
Thiazolidinediones	1.42 (1.06–1.90) *	1.42 (1.06–1.89) *	1.07 (0.76–1.51)	0.98 (0.69–1.41)
Meglitinides	1.91 (1.54–2.37) ***	1.90 (1.54–2.36) ***	1.07 (0.83–1.38)	1.01 (0.77–1.31)

Model 1 adjusted age and gender; Model 2 adjusted model 1 and comorbidities (hypertension, hyperlipidemia, cerebral vascular disease, coronary artery disease, chronic kidney disease, peripheral artery disease, heart failure, glomerulonephritis, STEMI, NSTEMI and unstable angina); Model 3 adjusted model 2 and medications (SGLT2i, DPP4i, GLP-1 agonist, insulin, alpha glucosidase inhibitors, sulfonylureas, thiazolidinediones, meglitinides, statin, aspirin, ACEI and ARB); *p* value < 0.05: *; *p* value < 0.01: **; *p* value < 0.0001: ***.

**Table 3:** Subgroup Analysis of the Impact of SGLT2i on 30-Day Dialysis Risk in Frequency-Matched T2DM patients.

	OR (95% CI)	Interaction *p*
Hypertension		0.1661
No	1.42 (0.23–8.69)	
Yes	0.20 (0.07–0.60) **	
Hyperlipidemia		0.399
No	0.55 (0.17–1.74)	
Yes	0.16 (0.03–0.73) *	
Cerebral Vascular Disease		0.8429
No	0.29 (0.11–0.76) *	
Yes	-	
Coronary Artery Disease		0.6168
No	0.28 (0.09–0.93) *	
Yes	0.11 (0.02–0.70) *	
Chronic Kidney Disease		0.0728
No	0.44 (0.14–1.44)	
Yes	0.18 (0.04–0.85) *	
Peripheral Artery Disease		0.9498
No	0.35 (0.15–0.82) *	
Yes	-	
Heart Failure		0.8668
No	0.27 (0.10–0.73) *	
Yes	-	
Glomerulonephritis		0.9631
No	0.28 (0.12–0.66) **	
Yes	-	
STEMI		0.9772
No	0.33 (0.14–0.76) **	
Yes	-	
NSTEMI		0.9599
No	0.37 (0.16–0.87) *	
Yes	-	
Unstable Angina		0.9598
No	0.41 (0.18–0.96) *	
Yes	-	

Model adjusted age, gender, comorbidities (hypertension, hyperlipidemia, cerebral vascular disease, coronary artery disease, chronic kidney disease, peripheral artery disease, heart failure, glomerulonephritis, STEMI, NSTEMI and unstable angina) and medications (DPP4i, GLP-1 agonist, insulin, metformin, alpha glucosidase inhibitors, sulfonylureas, thiazolidinediones, meglitinides, statin, aspirin, ACEI and ARB); *p* value < 0.05: *; *p* value < 0.01: **.

## Data Availability

The datasets generated and analyzed during this study are available from the corresponding authors upon reasonable request and subject to privacy and ethical restrictions.

## Supplementary Materials

The following supporting information can be downloaded at: https://www.mdpi.com/article/10.3390/ijms27041684/s1.

## Abbreviations

The following abbreviations are used in this manuscript:

AKI	Acute kidney injury
ASC	Apoptosis-associated speck-like protein containing a CARD
BUN	Blood urea nitrogen
CAD	Coronary artery disease
CIAKI	Contrast-induced acute kidney injury
CKD	Chronic kidney disease
CVD	Cerebrovascular disease
DPP4i	Dipeptidyl peptidase-4 inhibitor
ESRD	End-stage renal disease
GLP-1	Glucagon-like peptide-1
H&E	Hematoxylin and eosin
IL-1β	Interleukin-1 beta
IHC	Immunohistochemistry
NGAL	Neutrophil gelatinase-associated lipocalin
NHIRD	National Health Insurance Research Database
NLRP3	NOD-like receptor family pyrin domain-containing 3
NSTEMI	Non–ST-elevation myocardial infarction
PCI	Percutaneous coronary intervention
SGLT2i	Sodium-glucose co-transporter 2 inhibitor
STEMI	ST-elevation myocardial infarction
STZ	Streptozotocin
T2DM	Type 2 diabetes mellitus
