# Supplementary material for: SGLT2 Inhibitors Mitigate Contrast-Induced Acute Kidney Injury in Diabetes: Clinical and Experimental Evidence
Source: Int J Mol Sci. 2026 Feb 9;27(4):1684. doi: 10.3390/ijms27041684 (PMC12941132; doi:10.3390/ijms27041684)
Supplement: Supplementary file 1 [file ijms-27-01684-s001.zip › ijms-4105348-supplementary.pdf]

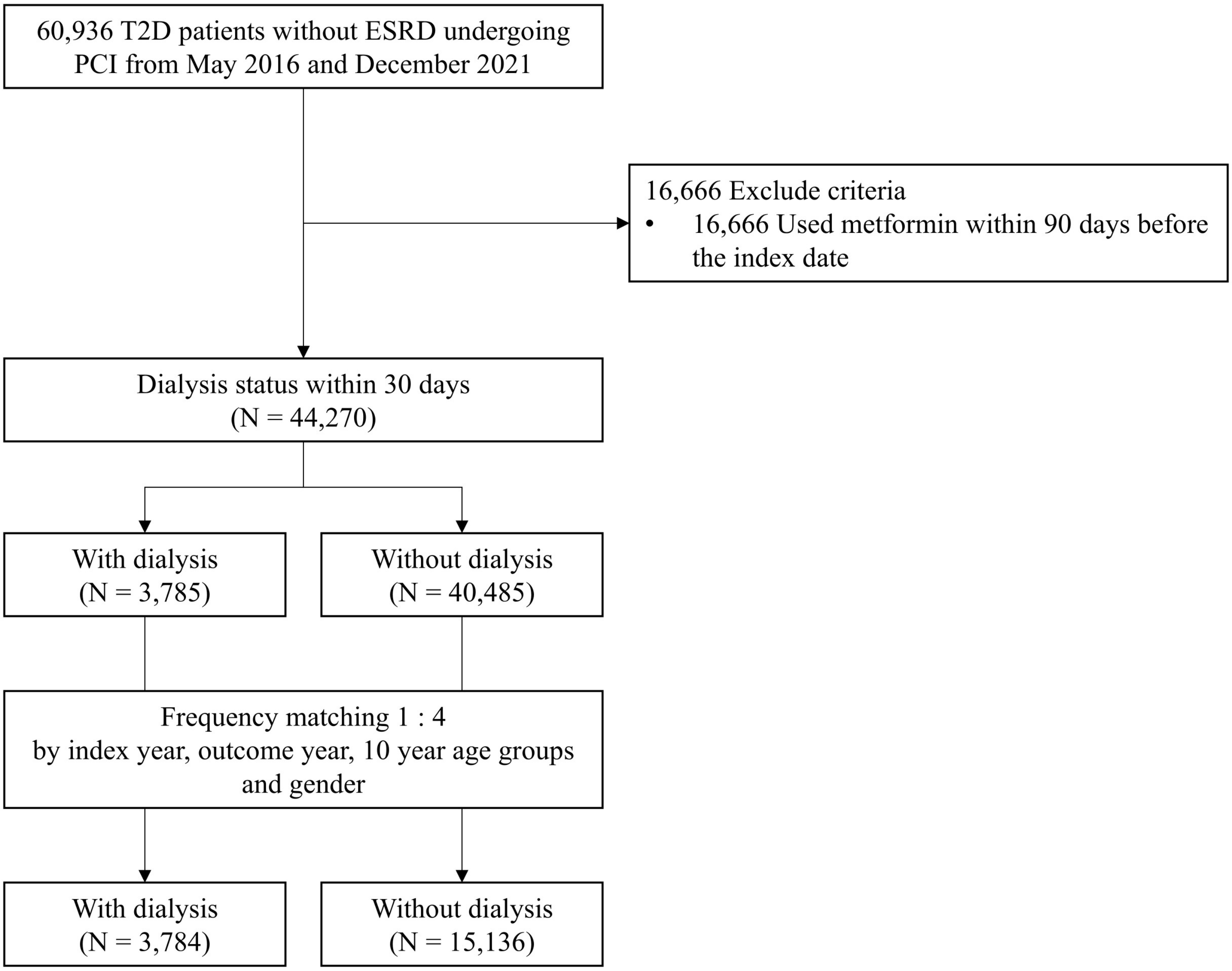

Figure S1. Flowchart of Cohort Construction and Matching for T2DM Patients Undergoing PCI.

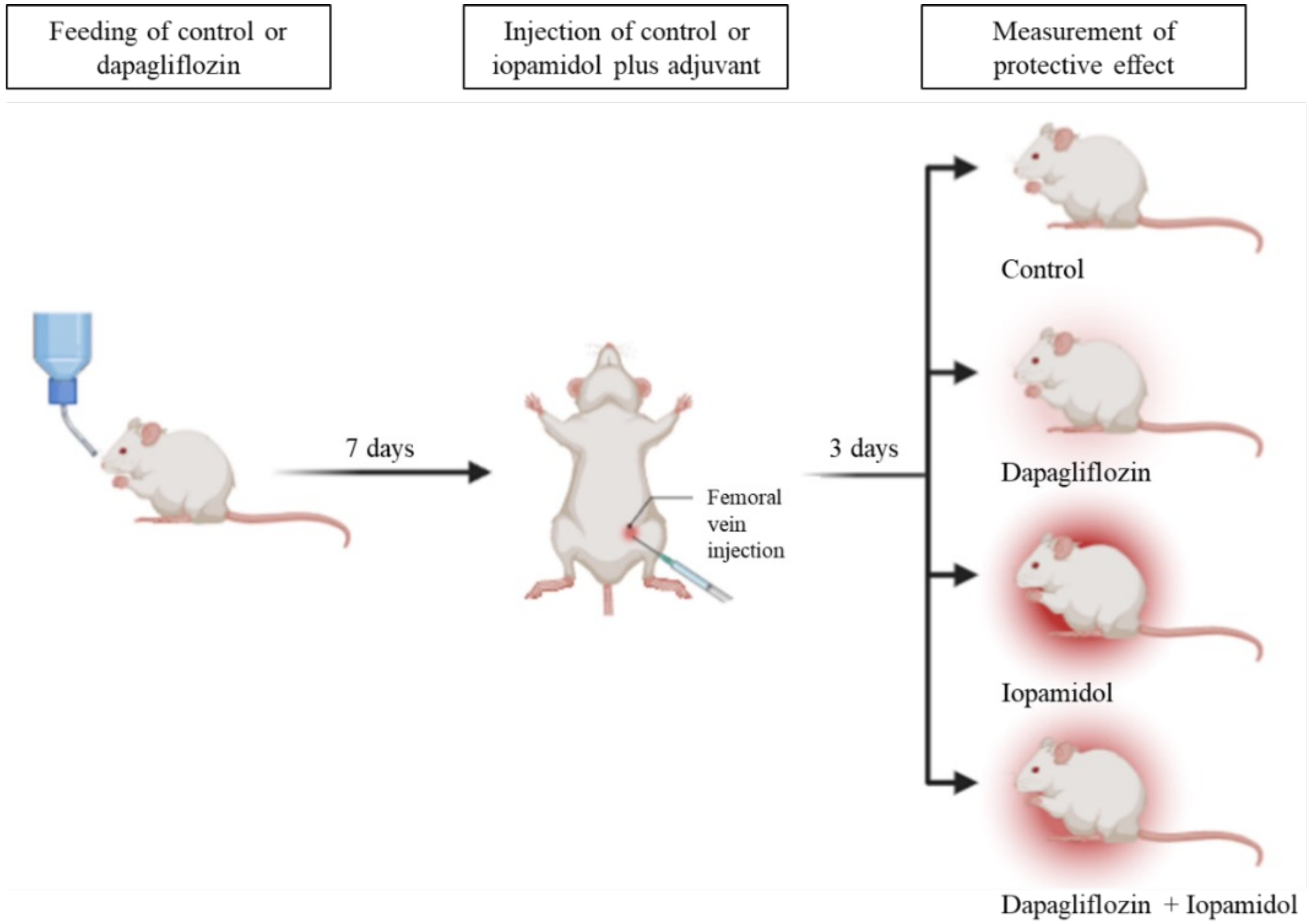

Figure S2. Experimental Design of the Diabetic Rat Model for Contrast-Induced Acute Kidney Injury.

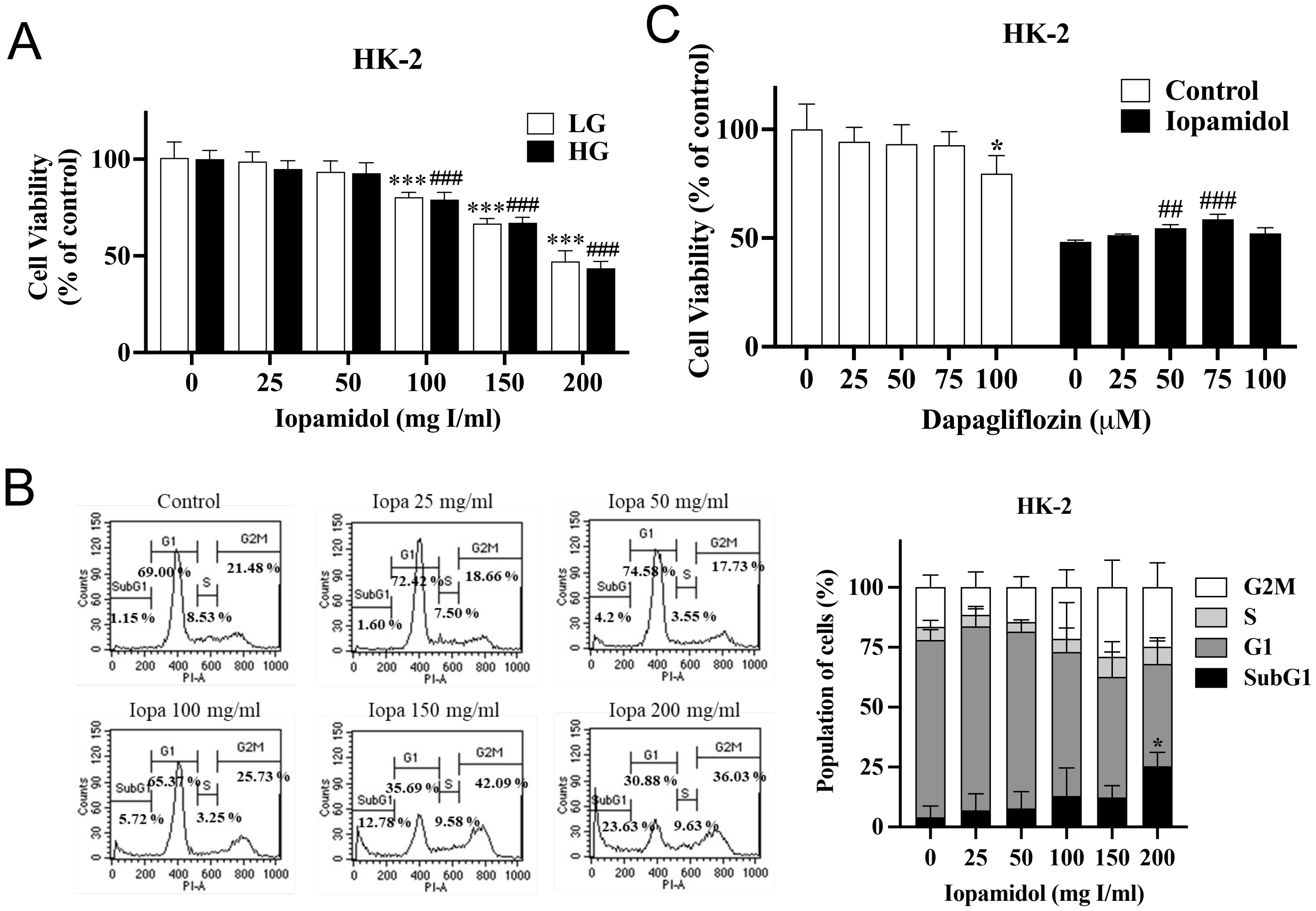

Figure S3. Dose-Dependent Effects of Iopamidol and Dapagliflozin on HK-2 Cell Viability and Cytotoxicity. (A) Cell viability assay of HK-2 cells treated with increasing concentrations of iopamidol under low glucose (LG, 5.5 mM) and high glucose (HG, 25 mM) conditions. (B) Flow cytometry analysis of cell cycle distribution in HK-2 cells treated with different concentrations of iopamidol under high glucose conditions. Representative histograms and corresponding quantification of the cell cycle phases (G2/M, S, G1, and SubG1) are shown. (C) Cell viability assay of HK-2 cells treated with increasing concentrations of dapagliflozin in the presence or absence of iopamidol under high glucose conditions. All experiments were performed at least three times independently. Statistical analysis was conducted using one-way ANOVA followed by post hoc tests. Data are presented as mean ± standard deviation (SD). Statistical significance is represented as $p < 0.05$, $p < 0.01$, and $p < 0.001$ versus the control group, and ##$p < 0.01$, ###$p < 0.001$ versus the Iopamidol-treated group. Scale bars are indicated in the images.

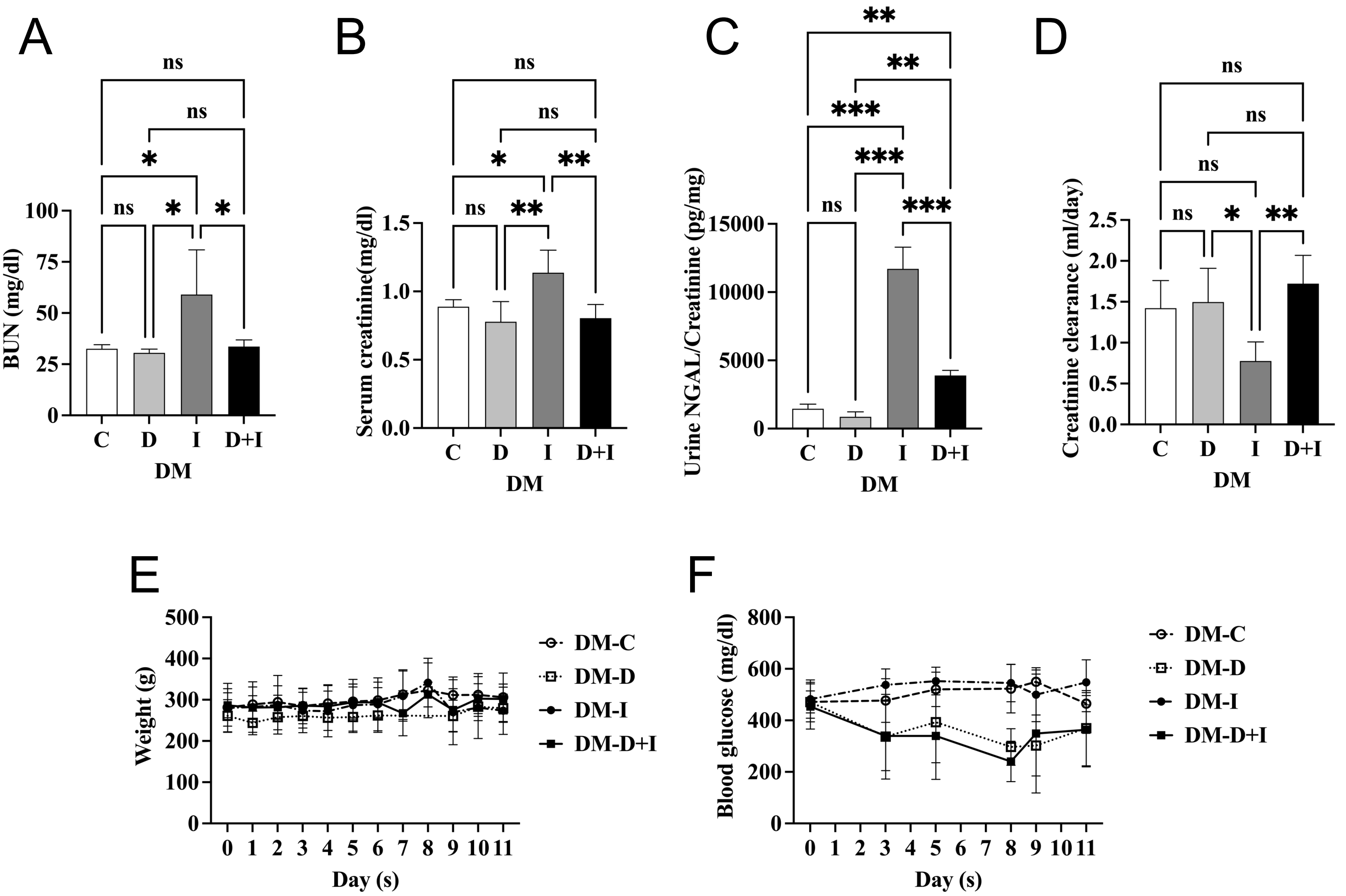

Figure S4. Effects of Dapagliflozin on Renal Function, Urinary NGAL, Body Weight, and Blood Glucose in Diabetic Rats with CIAKI. (A) BUN levels in different experimental groups. (B) Serum creatinine levels in different experimental groups. (C) Urine NGAL/creatinine ratio in different experimental groups. (D) Creatinine clearance in different experimental groups. (E) Body weight changes over time in different experimental groups. (F) Blood glucose levels over time in different experimental groups. All experiments were performed at least three times independently. Statistical analysis was conducted using one-way ANOVA followed by post hoc tests. Data are presented as mean ± standard deviation (SD). Statistical significance is represented as $p < 0.05$, $p < 0.01$, and $p < 0.001$. "ns" indicates no significant difference.
